# Galectin-3 level and the severity of cardiac diastolic dysfunction using cellular and animal models and clinical indices

**DOI:** 10.1038/srep17007

**Published:** 2015-11-19

**Authors:** Cho-Kai Wu, Mao-Yuan Su, Jen-Kuang Lee, Fu-Tien Chiang, Juey-Jen Hwang, Jiunn-Lee Lin, Jin-Jer Chen, Fu-Tong Liu, Chia-Ti Tsai

**Affiliations:** 1 Division of Cardiology, Department of Internal Medicine, National Taiwan University College of Medicine and Hospital, Taipei, Taiwan; 2 Graduate Institute of Clinical Medicine, College of Medicine, National Taiwan University, Taipei, Taiwan; 3 Department of Medical Imaging, National Taiwan University Hospital, Taipei, Taiwan; 4 Department of Internal Medicine and graduate institute of Clinical medical science, China Medical University, Taichung, Taiwan; 5 Institute of Biomedical Sciences, Academia Sinica, Taipei, Taiwan

## Abstract

Heart failure with preserved ejection fraction (HFPEF) is characterized by myocardial interstitial fibrosis. A total of 146 patients with HFPEF, were recruited. HFPEF severity was determined using Doppler imaging (E/Em) and also cardiac magnetic resonance imaging (CMRI). Canine modeling of HFPEF was induced by aortic banding. Hemodynamic and echocardiographic data were obtained before and after pressure loading and myocardial Galectin-3 was determined. Mechanical stretch of cultured cardiomyocytes served as the cellular model of HFPEF. Patients with severe HFPEF had significantly higher plasma Galectin-3 levels. Significant correlation between plasma Galectin-3 and E/Em in advanced HFPEF patients was noted. After 2 weeks of pressure overload in canine models, the protein expression of Galectin-3 from LV myocardial tissue was significantly increased (p < 0.01) compared with controls. Galectin-3 expression paralleled the severity of LV diastolic dysfunction by evaluation of CMRI (r = −0.58, p = 0.003) and tissue fibrosis (r = 0.59, p = 0.002). After adjusting for confounders for diastolic dysfunction, Galectin-3 levels were still associated with diastolic parameters both in humans (p < 0.001) and canine model (p = 0.041). Mechanical stretch increased Galectin-3 secretion in cultured cardiomyocytes. Both plasma and myocardial Galectin-3 levels correlated with severity of cardiac diastolic dysfunction.

Heart failure with preserved ejection fraction (HFPEF) has become an increasing concern in recent years. Studies suggest that at least one-third of patients with congestive heart failure actually have HFPEF^1^.

The symptoms and morbidity associated with HFPEF are similar to systolic heart failure^2^. In order to differentiate HFPEF from systolic heart failure, a new consensus was proposed^3^. Although N-terminal of the prohormone brain natriuretic peptide (NT-proBNP) was incorporated in the consensus as a diagnostic cytokine, its level fluctuated in HFPEF patients and often fell below the cut-off value when applied to large cohorts^4^. Brouwers *et al.* evaluated 8592 patients and concluded that risk factors such as age, history of atrial fibrillation or even cystatin C level were even better associated with the risk of HEPEF than NT-proBNP^5^ Another study also found that NT-proBNP was much lower in HFPEF group than in the systolic HF group^6^. Recently, Edelmann, *et al.* also showed that plasma Galectin-3 level are elevated in patients with stable HFpEF and relate to functional performance and quality of life but not NT-proBNP^7^.

Several new biomarkers, including Galectin-3, have been used for the diagnosis of HFPEF in recent years^8^. Galectin-3 is secreted by activated macrophages and modulates several physiological and pathological processes that are associated with the development of HFPEF, including inflammation and fibrosis^9^. Up-regulation of myocardial Galectin-3 has been demonstrated in animal models of pressure overload which are prone to HFPEF and a recent study also suggested that plasma Galectin-3 levels correlated with echocardiographic diastolic function parameters^10^. De Boer *et al.* followed a cohort of heart failure (HF) patients and compared the value of Galectin-3 in predicting the different types of HF. The authors concluded that Galectin-3 was an independent marker for outcome in HF patients and appeared to be particularly useful in HFPEF patients^11^.

Although the regulation of Galectin-3 has been extensively studied, the expression of Galectin-3, as it relates to hemodynamic changes, has not yet been described. In addition, the role of Galectin-3 in the diagnosis and differentiation of HFPEF has not been determined. We developed a canine model of HFPEF induced by aortic banding to compare hemodynamic changes with cardiac Galectin-3 expression. We also tested whether cardiomyocytes secrete Galectin-3 after stretch stimulation using an *in vitro* cellular model. In order to further elucidate the role of Galectin-3 in HFPEF patients, patients from the Taiwan Diastolic Heart Failure Registry (TDHFR) were enrolled and their plasma Galectin-3 levels were correlated with echocardiographic DHF severity. We hypothesized that both plasma and myocardial Galectin-3 levels would correlate with severity of DHF. Because CMRI (cardiac magnetic resonance imaging) T1 mapping along with ECV (extracellular volume fraction) quantification are promising modalities for noninvasive evaluation of diffuse myocardial fibrosis, we assessed the correlation between ECV and plasma Galectin-3 levels in current study.

## Materials and Methods

### Human model of diastolic heart failure

#### Taiwan Diastolic Heart Failure Registry

This study was performed in accordance with the Declaration of Helsinki and was approved by the institutional review board of the National Taiwan University Hospital (NTUH-REC No. 20070313R), and all subjects provided their written informed consent prior to participation in the study. The study group consisted of heart failure patients admitted to the cardiovascular ward of National Taiwan University Hospital from July 2007 to March 2011. Patients with the diagnosis of DHF (as defined in previous reports as well as by the recent consensus statement of the European Society of Cardiology) were enrolled in TDHFR^12^,^13^,^14^. In brief, diastolic heart failure (DHF) was defined as: (1) heart failure on the basis of Framingham criteria and normal systolic function (ejection fraction ≥50%); and (2) echocardiographic evidence of left ventricular diastolic dysfunction.

Finally, 146 patients with DHF (56 men and 90 women) were included in the current study. All participants received echocardiographic examinations as well as blood sampling for the estimation of plasma NT-proBNP and Galectin-3 levels. Using tissue Doppler imaging results and the recommendations of American society of echocardiography^15^, patients were divided into a severe DHF group (E/Em ≥ 15) vs. mild DHF group (15 > E/Em ≥ 8) (Table 1). We also describe the distribution of mild and severe DHF of our current population by other criteria in supplemental Table 1. The detailed inclusion and exclusion criteria are listed in the online data supplement in Supplemental Text 1.

#### Measurements of plasma Galectin-3 levels

All blood samples were collected with each patient after 12 h of fasting. Plasma Galectin-3 was measured with high-sensitivity enzyme-linked immunosorbent assay (ELISA)(catalog no. DGAL30, R&D Systems, Inc 614 McKinley Place NE Minneapolis, MN 55413, USA). Further detailed methods are provided in Supplemental Text 1.

#### Myocardial fibrosis assessed by cardiac magnetic resonance contrast-enhanced T1 mapping

**Figure 1:**
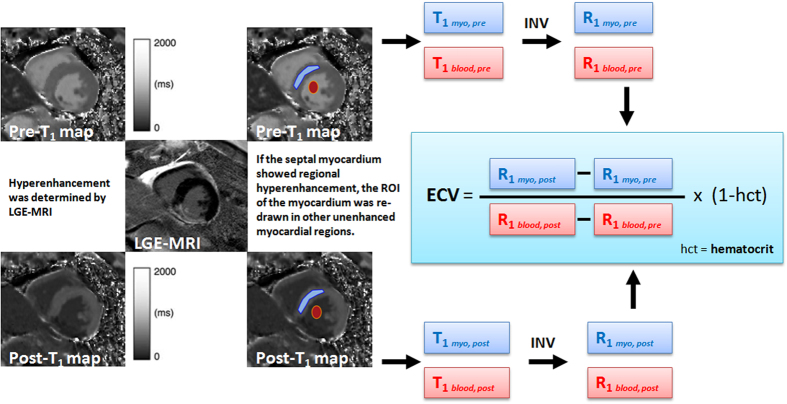
Extracellular volume (ECV) fraction quantification by T1 maps in a DHF patient with diffuse myocardial fibrosis.

We performed CMRI on 35 randomly selected subjects (25 patients with DHF, 10 control patients), using a clinical 3.0-T CMRI scanner (Trio, Siemens, Erlangen, Germany), as described previously^16^. The ECV was calculated and each ECV value was averaged over five short-axis slices for each subject (Figure 1). Further detailed methods are provided in Supplemental Text 1. Figure 1 shows the ECV quantification of a DHF patient with diffuse myocardial fibrosis.

#### Image Analysis for Left Ventricular Systolic Function and Diastolic Function

LV diastolic and systolic function were calculated according to previous study^17^. Briefly, endocardial and epicardial contours of the LV were determined at each slice level on cine MRI and the area enclosed by each contour was computed. From the interpolated curve of dV/dt, systolic and diastolic functional indices were determined at the minimal and maximal values as peak ejection rate (PER) and peak filling rate (PFR), respectively. A representative description for the PER and PFR is shown in supplemental Figure 3. Image analysis was performed using software developed in-house provided by Matlab 7.9 (Mathworks, Inc., Natick, MA, USA). Further detailed methods are provided in Supplemental Text 1.

### *In vivo* animal model of diastolic dysfunction

#### Canine model of LV diastolic dysfunction

Use of animals adhered to the NIH guidelines for the care and use of laboratory animals; protocols were approved by the Institutional Animal Care and Use Committee. Twelve dogs between 1 and 2 years of age, of either sex, were used in the experiments. Nine dogs were assigned to the aortic banding group, while the others comprised the sham-operated control group. The baseline body weight of each dog was measured, and the dogs were anesthetized with 0.15 mL/kg fentanyl-droperidol, intubated, and ventilated with nitrous oxide and oxygen (1:3 ratio) before surgery. Anesthesia was maintained by sufentanyl (0.15 mg/kg·min) and 1% isoflurane.

Thoracic aortic banding was performed, as previously described, to induce LV diastolic dysfunction due to chronic pressure overload^18^. Further detailed methods are provided in Supplemental Text 1.

#### Galectin-3 protein expression in the canine model

Cardiac tissue samples were homogenized in 50 mmol/L HEPES (pH 7.5), 150 mmol/L NaCl, 5 mmol/L EDTA, and protease inhibitors. Cell debris was removed by centrifugation for 2 min at 12,000 g, and protein concentration was determined with the Bradford reagent (Bio-Rad Laboratories, Hercules, CA, USA). Extracts were normalized to equal protein amounts and separated by SDS-PAGE. Galectin-3 protein concentrations were determined by western blot analysis with a GADPH control.At the beginning and end of the protocol, a complete echocardiographic study, including transthoracic echocardiography, was performed under anesthesia (Sonos 7500; Hewlett-Packard, Andover, MA, USA). Further detailed methods are provided in Supplemental Text 1.

### *In vitro* cellular model of pressure overload

#### Cell culture and *in vitro* stretch

HL-1 myocytes were cultured in Claycomb medium (JRH Bioscience, Lenexa, KS, USA) supplemented with 10% fetal bovine serum and maintained in a humid 10% CO_2_ incubator at 37 °C, as previously described^12^. *In vitro* mechanical stretch of cultured HL-1 myocytes was performed, as previously described^18^. HL-1 cardiomyocytes were then seeded (3 × 106 cells/well) onto 6-well collagen I-coated Bioflex plates (Flexcell International Corp, Hillsborough, NC) and cyclically strained via vacuum to 20% of elongation at a frequency of 1 Hz for 2, 6, or 24 hours. Further detailed methods are provided in Supplemental Text 1.

#### Galectin-3 concentration detection

Conditioned medium samples obtained from 12 strained samples were collected at the indicated times and frozen at −80 °C until assayed. Galectin-3 assessment was performed using ELISA (USCN Life Science & Technology Company).

### Statistical analysis

Data were analyzed using SPSS 15.0 software (SPSS Inc., Chicago, IL, USA). Continuous variables were represented as mean values ± standard deviation, while categorical variables were represented as frequencies. The association between categorical variables was tested by Pearson’s χ2 test. To test for normal distribution, the Kolmogorov-Smirnov test was applied. Comparisons between data showing normal distribution were performed using the Student’s t-test, or otherwise, by the Mann-Whitney U-test. The associations between cytokines and Doppler parameters or cytokines and CMRI diffuse fibrosis value were studied using Pearson’s correlation coefficient if the data met the criteria for normal distribution, or otherwise, by Spearman’s correlation test. Multiple linear regression modeling was applied followed by a forward stepwise analysis method to determine the factors associated with echocardiographic E/Em levels, including baseline, echocardiographic and laboratory parameters. Receiver operating characteristic (ROC) curves were used to assess the discriminative power of Galectin-3, NT-proBNP and LV mass/fibrosis for severe DHF. The area under the curve (AUC) was calculated. The optimal cutoff point for Galectin-3, defined as that with the minimum value of (1-sensitivity)^2^ + (1-specificity)^2^, or the shortest distance from the left upper corner to the ROC curve, was reported. Integrated discrimination improvement (IDI) of Galectin-3 for severe DHF was calculated by PredictABEL, R version 3.2.2 package (Netherlands) as previously reported^19^. A value of p < 0.05 was considered statistically significant for all tests.

## Results

### Human model of diastolic heart failure

#### Baseline characteristics of DHF patients

The baseline characteristics of mild vs. severe DHF patients are shown in Table 1. The baseline characteristics of both groups were comparable except for the type of medication administered. There were no significant differences in LV systolic function or left ventricular diameters between the two groups. Patients with severe DHF had larger sized atriums, longer mitral flow deceleration times, and larger E/Em ratios.

#### Plasma levels of Galectin-3 in DHF patients

**Figure 2:**
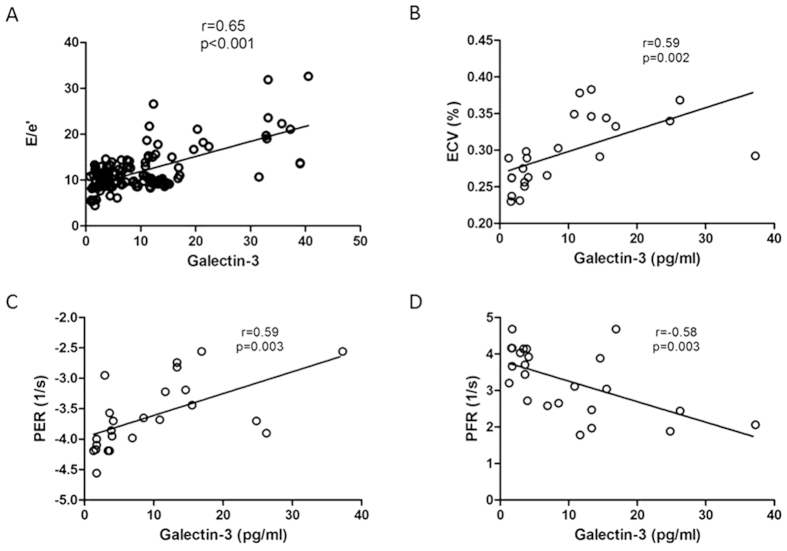
Human model of diastolic heart failure (DHF). (**A**) Correlations between plasma Galectin-3 levels with echocardiographic diastolic parameter (E/e’) in all DHF patients (**B**) Plasma Galectin-3 levels progressively increase concurrently with the ECV in the DHF groups, indicating good correlation between plasma Galectin-3 and severity of myocardial fibrosis. (**C**) Correlations between plasma Galectin-3 levels with peak ejection rate (PER), and (**D**) peak filling rate (PFR) in DHF patients.

Plasma Galectin-3 levels were significantly higher in severe DHF patients compared with mild DHF patients (severe DHF, 19.4 ± 12.4 ng/mL; mild DHF, 6.8 ± 5.3 ng/mL; *p* < .001) (Table 1). Galectin-3 levels were significantly associated with DT and E/Em levels in the whole DHF group (r = 0.30, p = 0.001 and r = 0.65, P < 0.001, respectively) (Table 2) (Fig. 2A).

Linear regression analysis was then performed and all the factors that might influence echocardiographic E/Em levels were incorporated. After correcting for LV mass index, diabetes, age, gender, plasma NT-proBNP and prescribed drugs (ACE inhibitor or diuretics), plasma Galectin-3 levels still significantly correlated with E/Em levels (B = 0.195, p < 0.001; Table 3). We also did the ROC analyses for the development of mild or severe DHF. The area under curve value is 0.87, 0.64 and 0.82 for Galectin-3, NT-proBNP and LV mass/fibrosis, respectively (supplemental Fig. 2A–C). The addition of Galectin-3 to traditional risk factors resulted a significant IDI (10.8% [CI, 3.4% to 18.1%]; P = 0.003). The best cut point of Galectin-3 for mild/severe DHF was 10.68 ng/ml with high sensitivity (0.80) and moderate specificity (0.74). To focus on HFPEF patients with overload-derived diastolic dysfunction¸ we excluded DHF patients with additional diagnosis of coronary heart disease (n = 28) and repeated correlation and linear regression analysis. The results were similar to the entire cohort which revealed that Galectin-3 levels were significantly associated with E/Em (r = 0.549, p < 0.001). Plasma Galectin-3 levels still significantly correlated with E/Em levels after adjustment for confounding factors. (B = 0.239, p < 0.001).

#### Relationship of myocardium fibrosis to plasma Galectin-3 level

Plasma Galectin-3 levels progressively increased concurrently with the ECV in the DHF groups (Fig. 2B) (r = 0.59, p = 0.002), indicating good correlation between plasma Galectin-3 levels and the severity of myocardial fibrosis. We performed correlation analysis between plasma Galectin-3 and LV functional index. Galectin-3 level was significantly correlated with PER (r = 0.59, p = 0.003), and PFR (r = −0.58, p = 0.003) (Fig. 2C,D).

### Animal model of LV diastolic dysfunction

#### Myocardial Galectin-3 expression was elevated in aortic banding group

**Figure 3:**
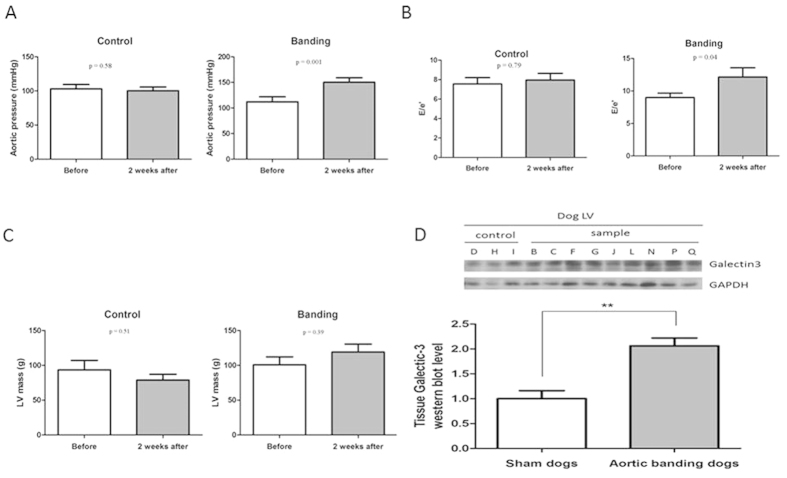
*In vivo* animal model of diastolic dysfunction. (**A**) After 2 weeks of aortic banding, the aortic blood pressure increases significantly. (**B**) The echocardiographic parameter for diastolic function (E/e′) is approximately 1.5-fold higher compared with controls, indicating that aortic banding induced LV diastolic dysfunction. (**C**) Calculated LV mass also increases significantly. (**D**) After 2 weeks of aortic banding, the tissue Galectin-3 also increases significantly. Cropped western blots were compared between controls and aortic banding animals for Galectin-3 protein concentrations. All the gels have been run under the same experimental conditions. Full-length blots are included in Supplemental Figure 1.

After 2 weeks of aortic banding, the aortic blood pressure increased significantly after the aortic banding (Fig. 3A). The echocardiographic parameter for diastolic function (E/e′) was approximately 1.5-fold higher compared with controls, indicating that the aortic banding induced LV diastolic dysfunction (Fig. 3B). To test whether local Galectin-3 expression was an early and reliable marker for the severity of LV diastolic dysfunction (as observed in the clinical study), the myocardial expression of Galectin-3 was measured. After aortic banding, the tissue Galectin-3 increased significantly after 2 weeks (Fig. 3D). As in the severe DHF patients, tissue Galectin-3 correlated with the echocardiographic diastolic parameter, E/Em. After adjusting for LV mass, aortic pressure, and LV ejection fraction by multiple linear regression method, tissue Galectin-3 still significantly correlated with echocardiographic E/Em ratios (B = 7.6, p = 0.041; Table 4).

### Cell model of LV pressure overload

#### Cellular Galectin-3 expression after mechanical stretch

**Figure 4:**
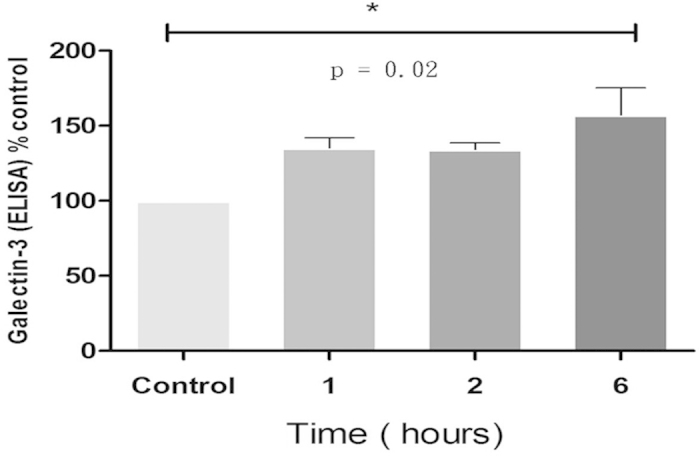
*In vitro* cellular model of pressure overload. When compared with the control cardiomyocytes, stretched cardiomyocytes exhibit a 32% increase in Galectin-3 secretion into the culture medium after 6 h of stretch (*p < 0.05).

*In vitro* mechanical stretch of cardiomyocytes was used to mimic the *in vivo* myocardial pressure overload involved in the pathogenesis of diastolic dysfunction. Compared with control cardiomyocytes, stretched cardiomyocytes exhibited a significant increase in Galectin-3 secretion into the culture medium. Further analysis showed a 32% increase in Galectin-3 secretion after 6 h of stretch when compared with control cardiomyocytes (p = 0.02; Fig. 4).

## Discussion

Based on CMRI results, the level of plasma Galectin-3 correlated with the severity of LV diastolic dysfunction as well as the severity of cardiac fibrosis. In addition, plasma Galectin-3 was significantly associated with echocardiographic parameters for diastolic dysfunction, especially in advanced HFPEF patients. Accordingly, in the animal model, we also showed that cardiac Galectin-3 increased significantly as early as 2 weeks after pressure overload. Moreover, the expression of Galectin-3 paralleled the severity of LV diastolic dysfunction. In the cellular model, cardiomyocytes produced and secreted more Galectin-3 after mechanical stretch (mimicking pressure overload stimulation). These results implied that Galectin-3 level may directly reflect changes in LV diastolic function or cardiac fibrosis and may serve as a sensitive marker to monitor the effect of treatment.

In the present study, the level of Galectin-3 increased after myocardial stretch. In our previous study, myocardial stretch was also associated with upregulation of connective tissue growth factor (CTGF) which may be the downstream messenger of Galecin-3^20^. CTGF could be an important intermediary for sensing stimulation and promoting further fibrosis cascades which in turn lead to the development of HFPEF. In addition, Galectin-3 plays an important role in the inflammatory response, which is important in cardiac remodeling^21^. Galectin-3 has also been convincingly linked to cardiac fibrosis and damage^22^. Systemic inflammation may cause cardiac diastolic dysfunction by decreasing diastolic calcium re-uptake through downregulation of *Sarcoplasmic reticulum Ca2+-ATPase (SERCA)* gene expression^12^. SERCA is one of the most extensively studied calcium channels with respect to diastolic dysfunction. Decreased activity of the SERCA pump slows the removal of calcium from the cytosol, which impairs the diastolic relaxation of contractile proteins^17^. It is logical to speculate that through augmentation of the inflammatory process, Galectin-3 may lead to down-regulation of SERCA and cardiac diastolic dysfunction.

Previous studies have shown that the change of myocardial expression of both Galectin-3 mRNA and protein paralleled the change of plasma Galectin-3 level^23^,^24^. However, The source of increasing cardiac Galectin-3 in various cardiovascular diseases remains unclear. In the present study, we measured Galectin-3 concentration in pure cardiomyocytes (cell model), intact heart tissue (animal model), and plasma (human study). We found that Galectin-3 increased significantly even after only a slight increase in blood pressure (animal model) or after short-term stretching (cell model). These results strengthen the hypothesis that cardiomyocytes could respond to the stimulation of elevated blood pressure or mechanical load change by secreting Galetin-3, which in turn could lead to paracrine responses, e.g., up-regulation of fibrosis or inflammation. In addition, the results of our human study were also consistent with our animal and cellular models in that the concentration of Galectin-3 was significantly higher in severe HFPEF patients compared with mild HFPEF patients. After adjusting for the influence of plasma pro-BNP level, Galectin-3 remained an independent factor for cardiac diastolic dysfunction. The correlation between cardiac diastolic function indicators was stronger for Galectin-3, compared with pro-BNP, in our population. Therefore, Galectin-3 may be useful for early detection, phenotyping, risk stratification, and therapeutic targeting of individuals with early or established HFPEF^11^.

**Figure 5:**
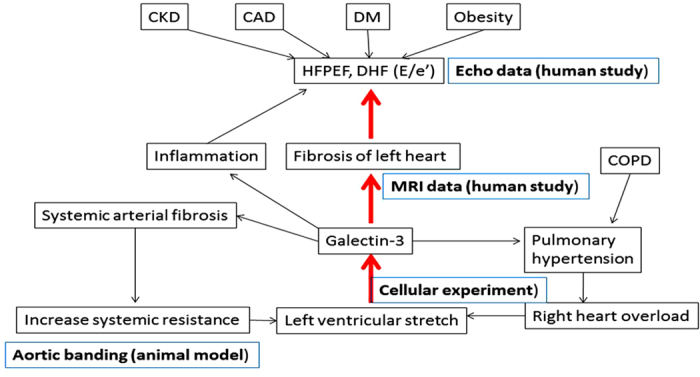
Schematic representations for the etiology of diastolic heart failure (DHF) and the multi-face influences of Galectin-3 towards the disease. HFPEF, heart failure with preserved ejection fraction; COPD, chronic obstructive pulmonary disease; CKD, chronic kidney disease; CAD, coronary artery disease; DM, diabetes mellitus; MRI, magnetic resonance imaging.

Several studies have addressed the prognostic value of Galectin-3 in patients with either systolic heart failure or HFPEF. One recent study measured plasma Galecin-3 levels repeatedly at baseline and after serial follow-up in two large cohorts of patients with either chronic or acute decompensated heart failure. The authors concluded that Galectin-3 level provided important and significant prognostic value in patients with chronic or acute decompensated heart failure^25^. We found that Galectin-3 levels not only reflect the severity of cardiac diastolic dysfunction and fibrosis (in our human study) but are also sensitive to shearing force or pressure changes. Therefore, modern therapies for HFPEF that target only downstream factors (e.g., fibrosis and renin-angiotensin aldosterone systems) may not be able to decrease plasma Galectin-3 levels. Targeting Galectin-3 may be an upstream therapeutic option for the treatment of all types of heart failure. There is still much uncertainty regarding the development of a therapy which can target Galectin-3 directly. First, we do not know how Galectin-3 is regulated at the transcriptional and translational levels in the heart. Previous mechanistic studies performed on cardiac fibroblasts and macrophages have shown that the TGF-β/Smad pathway was involved^26^. Although inflammatory signals also contribute to the regulation of Galectin-3, the signals or cytokines which govern the production and secretion of Galectin-3 remain enigmatic, warranting future explorative pharmacological studies. The etiology of HFPEF are multiple and complex. In our current studies, we tried to prove one of the possible mechanisms (our central hypothesis) that myocardium could secret Galectin-3 under the stimulation of myocardial stretch (or pressure overload) and the secreted Galectin-3 (in plasma or within myocardium) may in turn trigger myocardial fibrosis, which results in diastolic dysfunction or HFPEF (see Fig. 5 for the schematic presentation of our central hypothesis). To prove this hypothesis, single human studies might not be adequate, and supplementary animal model is mandatory. We therefore adopted an aortic-banding canine model with aortic banding that has been a well-documented myocardial pressure-overload model. Finally, although we had proven that myocardial Galectin-3 was increased after pressure overload, we did not know the source of increased Galectin-3 (fibroblasts or cardiomyocytes). Furthermore, the response to aortic band may be diverse in different animals. Therefore, we further used a pure cellular model to prove that single stretch per se could stimulate the cultured cardiomycytes to secret Gaelectin-3.

Our study had several limitations. We did not directly measure the tissue Galectin-3 expression in patients with HFPEF. Theoretically, this is not feasible in human studies. In addition, our animal model only demonstrated pressure overload-induced LV diastolic dysfunction. There are other mechanisms of LV diastolic dysfunction, such as myocardial ischemia. The role of Galecin-3 in ischemia-induced or other causes of LV diastolic dysfunction is less studied. We did not measure plasma Galecin-3 levels in our canine model and we did not perform CMRI in all recruited patients. In addition, there are more structural, functional, and molecular biological evidence supports that DHF is a distinct syndrome. The relative wall thickness and LV mass index seemed showed a trend of increase in patients with severe DHF. We recognized from numerous previous studies that DHF is a distinct form of HF other than systolic HF. Structurally, most patients with systolic HF have eccentric LV hypertrophy, whereas those with DHF have concentric LV hypertrophy^27^. In our current study, the relative wall thickness and the severity of concentric hypertrophy increased as the disease progressed. The concentric LV hypertrophy or concentric remodeling associated with DHF is frequently caused by chronic pressure overload (e.g. hypertension). In addition to abnormal relaxation due to increased LV stiffness, concentric LV remodeling may activate the calcineurin/nuclear factor pathways of activated T cells (NFAT) pathway and result in the increased expression of fetal β-myosin heavy chain and downregulation of sarcoplasmic reticulum^28^. Therefore, the severity of LV remodeling is also a factor to determine the severity of DHF in our patients. In patients with diabetes mellitus, an increasing rate of diastolic dysfunction was noted which could be explained by the infiltration of advanced glycation end products^29^. Higher BMI, or visceral adipose tissue amount could also lead to subclinical LV diastolic dysfunction by low-grade inflammation or even accumulation of epicardial fat^30^,^31^. We did not control renal function in our current study. However, chronic kidney disease is associated with fluid overload, higher prevalence of hypertension and LV hypertrophy which contribute to formation of LV diastolic dysfunction^32^. Finally, pulmonary hypertension or right ventricular dysfunction is highly prevalent and often severe in HFPEF^33^. We did not obtain the data of pulmonary artery systolic pressure (PASP) and were not able to analysis the role between PASP and Galectin-3.

In conclusion, we found a significant correlation between plasma and tissue Galectin-3 levels and the degree of diastolic dysfunction and severity of myocardial fibrosis. We also demonstrated that both cellular and tissue Galecin-3 responded well to changes in stretching and pressure overload, respectively, indicating that Galecin-3 could be an important intermediary in promoting further fibrosis and/or inflammatory cascades. While there is little information regarding the medical management of HFPEF, novel therapies that downregulate the overexpression of Galectin-3 may indicate a new direction for HFPEF therapy. Targeting Galectin-3 may be an upstream therapeutic option for the treatment of all types of heart failure.

## Additional Information

**How to cite this article**: Wu, C.-K. *et al.* Galectin-3 level and the severity of cardiac diastolic dysfunction using cellular and animal models and clinical indices. *Sci. Rep.* **5**, 17007; doi: 10.1038/srep17007 (2015).

## Supplementary Material

Supplementary Information

## Figures and Tables

**Table 1:** Baseline Characteristics and Plasma Galectin-3 Levels in Severe and Minor DHF Patients.

	Control(n = 30)	Mild DHF(n = 112)	Severe DHF(n = 34)	p*
Age (years)	63.23 ± 9.04	68.57 ± 8.42	71.54 ± 9.52	0.15
Sex (M/F)	11/19	47/65	9/25	0.09
BMI (kg/m^2^)	25.9 ± 2.4	25.9 ± 3.2	26.8 ± 4.1	0.27
Diabetes mellitus (%)		24 (21)	14 (41)	0.11
Hyperlipidemia (%)		50 (44)	12 (38)	0.54
Blood pressure (mmHg)		142.2 ± 18.7	151.4 ± 24.5	0.02*
NYHA Fc		2.12 ± 0.43	2.83 ± 0.68	<0.001**
Antihypertensive therapy				
ACEI + ARB (%)		43 (38)	24 (71)	0.01**
β-Blocker (%)		72 (64)	26 (76)	0.09
CCB (%)		69 (62)	24 (71)	0.38
Nitrates (%)		11 (10)	8 (24)	0.11
Statin (%)		25 (22)	10 (30)	0.51
Diuretics (%)		60 (54)	30 (88)	0.01**
Aldactone		37 (33)	10 (29)	0.60
Echocardiographic data				
LAVI (ml/m^2^)		41.6 ± 4.5	48.1 ± 3.4	0.01*
LVEF (%)		67.6 ± 8.0	68.6 ± 5.2	0.35
LVEDVI (ml/m^2^)		71.5 ± 20.8	69.7 ± 14.9	0.66
LVEDD (mm)		45.3 ± 4.9	45.1 ± 5.1	0.86
LVESD (mm)		28.1 ± 4.5	27.7 ± 4.8	0.70
DT (cm/s)		247.3 ± 49.9	273.6 ± 97.1	0.05**
E/A		0.85 ± 0.30	0.94 ± 0.45	0.22
LVMI		187.78 ± 44.9	198.83 ± 47.8	0.25
Cytokine levels				
NT-proBNP (pg/mL)		183.9 ± 135.5	565.3 ± 472.9	0.003**
Galectin-3 (ng/mL)	3.4 ± 2.2	6.8 ± 5.3	19.4 ± 12.4	<0.001**

Continuous variables are represented as mean ± SD, while categorical variables are represented as frequencies.

Abbreviations: DHF, diastolic heart failure; NT-proBNP, N-terminal pro-brain natriuretic peptide; BMI, body mass index; NYHA, New York Heart Association; ACEI, angiotensin-converting enzyme inhibitor; ARB, angiotensin II type I receptor blocker; LAVI, left atrium volume index; LVEF, left ventricular ejection fraction; LVEDVI, left ventricular end-diastolic volume index; LVEDD, left ventricular end-diastolic dimension; LVESD, left ventricular end-systolic dimension; DT, mitral flow deceleration time; E/A, early mitral valve flow velocity (E) divided by late mitral flow velocity (A); E/e′, E divided by early diastolic (e′) lengthening velocities in tissue Doppler imaging; LVMI, left ventricular mass index; CTGF, connective tissue growth factor.

The p value denotes the significance between mild and severe DHF patients. *p < 0.05; **p < 0.005.

**Table 2:** Correlation between echocardiographic parameters of left ventricular diastolic function and plasma concentrations of Galectin-3 in DHF subjects.

All DHF patients (n = 146)		
	R	P
E/A	0.08	0.375
E/Em	0.65	<0.001*
DT	0.3	0.001*

Abbreviations: E, mitral valve ejection flow; A, mitral valve atrium flow; Em, peak mitral annular early diastolic velocity; DT, mitral valve ejection flow deceleration time; r, correlation coefficient;

*p < 0.05.

**Table 3:** Multivariable linear regression models for significant major determinants of echocardiographic E/Em level in the DHF cohorts.

Variables	B	SE	p
Age	0.104	0.034	0.02*
Gender	−1.231	0.585	0.037*
NYHA Fc	2.143	0.554	<0.001*
Plasma Galectin-3	0.195	0.033	<0.001*

Abbreviations: E/Em, E divided by early diastolic (Em) lengthening velocities in tissue Doppler imaging; NYHA Fc, New York Heart Association function class.

B and SE were estimated by forward stepwise multiple linear regression. *p < 0.05.

**Table 4:** Multivariable linear regression models for major determinants of echocardiographic E/e’ ratio in a canine model.

Variables	B	SE	P
LV mass	0.023	0.012	0.45
LV ejection fraction	−2.34	3.1	0.13
Aortic pressure	−0.034	0.022	0.09
Tissue Galectin-3	7.6	3.1	0.041*

Abbreviations: LV, left ventricular.

B and SE were estimated by multiple linear regression. *p ≤ 0.05.
