# Supplementary material for: Galectin-3 level and the severity of cardiac diastolic dysfunction using cellular and animal models and clinical indices
Source: Sci Rep. 2015 Nov 19;5:17007. doi: 10.1038/srep17007 (PMC4652206; doi:10.1038/srep17007)

Supporting Online Materials for

Galectin-3 level and the severity of cardiac diastolic dysfunction using cellular and animal models and clinical indices

Cho-Kai Wu^a,b^; Mao-Yuan Su^c^; Jen-Kuang Lee^a^; Fu-Tien Chiang^a^; Juey-Jen Hwang^a^; Jiunn-Lee Lin^a^; Jin-Jer Chen^d,e^; Fu-Tong Liu^e^; Chia-Ti Tsai^a^

**Materials and Methods**

***Human model of diastolic heart failure***

**Taiwan Diastolic Heart Failure Registry**

The study was approved by the institutional review board of the National Taiwan University Hospital (NTUH-REC No. 20070313R), and all subjects provided their written informed consent prior to participation in the study. The study group consisted of heart failure patients admitted to the cardiovascular ward of National Taiwan University Hospital from July 2007 to March 2011. Patients with the diagnosis of DHF (as defined in previous reports as well as by the recent consensus statement of the European Society of Cardiology) were enrolled in TDHFR.^1-3^ In brief, diastolic heart failure (DHF) was defined as: (1) heart failure on the basis of Framingham criteria and normal systolic function (ejection fraction ≥ 50%); and (2) echocardiographic evidence of left ventricular diastolic dysfunction.

Left ventricular diastolic dysfunction was defined as an increased ratio of early (E) mitral valve flow velocity divided by early diastolic (Em) lengthening velocity on tissue Doppler imaging (E/Em ≧ 15), or if 15 > E/Em ≧ 8, a mitral inflow E/ late (A) mitral valve flow velocity ratio < 0.5, with a deceleration time > 280 cm/sec units.

Patients who had chronic renal failure (defined as a creatinine level > 250 μmol/L), significant hepatic disease, secondary hypertension, pericardial disease, severe valvular heart disease, cancer, chronic obstructive pulmonary disease, or chronic atrial fibrillation were excluded. None of our patients has symptoms or signs suggestive of secondary hypertension (e.g. truncal obesity, [glucose intolerance](https://en.wikipedia.org/wiki/Glucose_intolerance), [moon face](https://en.wikipedia.org/wiki/Moon_face),weight loss with increased appetite, [fast heart rate](https://en.wikipedia.org/wiki/Tachycardia), localized abdominal [bruit](https://en.wikipedia.org/wiki/Bruit) to the left or right of the midline, decreased blood pressure in the lower extremities or abrupt episodes of hypertension accompanied by headache, [palpitations](https://en.wikipedia.org/wiki/Palpitation)). There is no patient with the diagnosis of resistant hypertension or young/late onset hypertension (below age of 39.) According to recent consensus and diagnosis algorism, we made the diagnosis of essential hypertension for all of our patients. Demographic data were collected from medical records. Plasma cholesterol, triglycerides, low density lipid, high density lipid, white blood cell counts, and creatinine levels were measured within 1 week of DHF diagnosis. Finally, 146 patients with DHF (56 men and 90 women) were included in the current study. All participants received echocardiographic examinations as well as blood sampling for the estimation of plasma NT-proBNP and Galectin-3 levels.

Using tissue Doppler imaging results and the recommendations of American society of echocardiography,^4^ patients were divided into a severe DHF group (E/Em ≥ 15) vs. mild DHF group (15 > E/Em ≥ 8). The concentration of plasma Galectin-3 and the correlation of Galectin-3 with LV diastolic function parameters were compared between the two groups.

**Measurements of plasma Galectin-3 levels**

All blood samples were collected after 12 h of fasting from the antecubital vein between 08:00 and 10:00 h, with each patient in the supine position. The blood was spun at 2000g for 15 min, and the plasma was separated and stored at -80°C until use. Plasma Galectin-3 was measured with high-sensitivity enzyme-linked immunosorbent assay (ELISA) (catalog no. DGAL30, R&D Systems, Inc 614 McKinley Place NE Minneapolis, MN 55413, USA) with a minimally detectable range from 0.003 to 0.085 ng/mL. The calculated overall interassay and intra-assay coefficients of variation were both < 6% for Galectin-3.

**Myocardial fibrosis assessed by cardiac magnetic resonance contrast-enhanced T1 mapping**

We performed cardiac magnetic resonance imaging (CMRI) on 35 randomly selected subjects (25 patients with DHF, 10 control patients), using a clinical 3.0-T CMRI scanner (Trio, Siemens, Erlangen, Germany), as described previously.^5^ Briefly, myocardial T1 maps were acquired before and 10 minutes after infusion of 0.1 mmole/kg of Gd-DTPA. Five short-axis planes were acquired to cover the entire left ventricle (LV). For T1 maps, the regions of interest (ROI) in the blood and the myocardium of the LV were segmented manually in the central area of the LV cavity and the septal myocardium for each slice, respectively. The extracellular volume fraction (ECV) was calculated using the ratio of the change in relaxation rate (1/T1) in the myocardium to that in the blood and multipled by (1- hematocrit )(Figure 1A). Each ECV value was averaged over five short-axis slices for each subject.

**Image Analysis for Left Ventricular Systolic Function and Diastolic Function**

LV diastolic and systolic function were calculated according to previous study.^5^ Briefly, endocardial and epicardial contours of the LV were determined at each slice level on cine MRI and the area enclosed by each contour was computed.^6^ LV volumes for each time point were then determined by the Simpson’s rule to obtain the volume-time curve of the LV. To obtain the LV diastolic dysfunction represented by rate of change in LV volume (dV/dt), we calculated the differential of the volume change (dV/dt) and performed interpolation with a cubic b-spline function with an interval of 1 ms. From the interpolated curve of dV/dt, systolic and diastolic functional indices were determined at the minimal and maximal values as peak ejection rate (PER) and peak filling rate (PFR), respectively. Image analysis was performed using software developed in-house provided by Matlab 7.9 (Mathworks, Inc., Natick, MA, USA).

***In vivo animal model of diastolic dysfunction***

**Canine model of LV diastolic dysfunction**

Use of animals adhered to the NIH guidelines for the care and use of laboratory animals; protocols were approved by the Institutional Animal Care and Use Committee.

Twelve dogs between 1 and 2 years of age, of either sex, were used in the experiments. Nine dogs were assigned to the aortic banding group, while the others comprised the sham-operated control group. The baseline body weight of each dog was measured, and the dogs were anesthetized with 0.15 mL/kg fentanyl-droperidol, intubated, and ventilated with nitrous oxide and oxygen (1:3 ratio) before surgery. Anesthesia was maintained by sufentanyl (0.15 mg/kg⋅min) and 1% isoflurane.

Thoracic aortic banding was performed, as previously described, to induce LV diastolic dysfunction due to chronic pressure overload.^7^ In brief, prolonged pressure overload was produced through direct aortic banding with an external balloon dilation catheter, and the balloon was inflated to produce an increase in LV systolic pressure of approximately 50 mm Hg immediately after the procedure. The balloon was inflated for 2 weeks. The animals (including controls) were then euthanized by anesthetic overdose, and the LV free walls were quickly harvested for analysis.

**Galectin-3 protein expression in the canine model**

Cardiac tissue samples were homogenized in 50 mmol/L HEPES (pH 7.5), 150 mmol/L NaCl, 5 mmol/L EDTA, and protease inhibitors. Cell debris was removed by centrifugation for 2 min at 12,000g, and protein concentration was determined with the Bradford reagent (Bio-Rad Laboratories, Hercules, CA, USA). Extracts were normalized to equal protein amounts and separated by SDS-PAGE. Galectin-3 protein concentrations were determined by western blot analysis with a GADPH control.

**Evaluation of canine cardiac diastolic function by echocardiography**

At the beginning and end of the protocol, a complete echocardiographic study, including transthoracic echocardiography, was performed under anesthesia (Sonos 7500; Hewlett-Packard, Andover, MA, USA). In brief, left atrial diameter, LV dimensions, and wall thickness were measured in the parasternal long axis with M-mode ultrasound. Conventional transmitral flow was measured in the apical 4-chamber view. The early (E) and atrial (A) transmitral peak flow velocities, E/A ratio, and E-wave deceleration time were measured. Pulsed wave tissue doppler imaging (TDI) was performed by activating the TDI function in the same machine with early diastolic (Em) velocities recorded from septal (or medial) and lateral aspects of the 4-chamber view.

***In vitro cellular model of pressure overload***

**Cell culture and *in vitro* stretch**

HL-1 myocytes were cultured in Claycomb medium (JRH Bioscience, Lenexa, KS, USA) supplemented with 10% fetal bovine serum and maintained in a humid 10% CO_2_ incubator at 37°C, as previously described.^1^ *In vitro* mechanical stretch of cultured HL-1 myocytes was performed, as previously described.^8,9^ In brief, the Flexercell FX-4000 Tension Plus (Flexcell International Corp, Hillsborough, NC) is a computer-based system which utilizes vacuum suction to strain cells seeded on flexible collagen I-coated electrometric membranes. The deformation of the elastomeric membrane causes the adherent cells to similarly deform which could represent pressure stimulation over cardiomyocytes. HL-1 cardiomyocytes were then seeded (3x 10^6^ cells/well) onto 6-well collagen I-coated Bioflex plates (Flexcell International Corp, Hillsborough, NC) and cyclically strained via vacuum to 20% of elongation at a frequency of 1 Hz for 2, 6, or 24 hours.

**Galectin-3 concentration detection**

Conditioned medium samples obtained from 12 strained samples were collected at the indicated times and frozen at -80°C until assayed. Galectin-3 assessment was performed using ELISA (USCN Life Science & Technology Company).

***Statistical analysis***

Data were analyzed using SPSS 15.0 software (SPSS Inc., Chicago, IL, USA). Continuous variables were represented as mean values ± standard deviation, while categorical variables were represented as frequencies. The association between categorical variables was tested by Pearson’s χ2 test. To test for normal distribution, the Kolmogorov-Smirnov test was applied. Comparisons between data showing normal distribution were performed using the Student’s t-test, or otherwise, by the Mann-Whitney U-test. The associations between cytokines and Doppler parameters or cytokines and CMRI diffuse fibrosis value were studied using Pearson’s correlation coefficient if the data met the criteria for normal distribution, or otherwise, by Spearman’s correlation test. We calculated the significance of the difference between the correlation coefficient of severe vs. mild DHF groups using

 the Fisher r-to-z transformation (<http://faculty.vassar.edu/lowry/rdiff.html>). Multiple linear regression modeling was applied followed by a forward stepwise analysis method to determine the factors associated with echocardiographic E/Em levels, including baseline, echocardiographic and laboratory parameters. A value of p < 0.05 was considered statistically significant.

**References**

1. Wu CK, Lee JK, Chiang FT, et al. Plasma levels of tumor necrosis factor-alpha and interleukin-6 are associated with diastolic heart failure through downregulation of sarcoplasmic reticulum Ca2+ ATPase. Crit Care Med 2011; 39:984-992

2. Wu CK, Tsai CT, Chang YC, et al. Genetic polymorphisms of the angiotensin II type 1 receptor gene and diastolic heart failure. J Hypertens 2009; 27:502-507

3. Wu CK, Luo JL, Tsai CT, et al. Demonstrating the pharmacogenetic effects of angiotensin-converting enzyme inhibitors on long-term prognosis of diastolic heart failure. Pharmacogenomics J 2010; 10:46-53

4. Nagueh SF, Appleton CP, Gillebert TC, et al. Recommendations for the evaluation of left ventricular diastolic function by echocardiography. Eur J Echocardiogr 2009; 10:165-193

5. Su MY, Lin LY, Tseng YH, et al. CMR-Verified Diffuse Myocardial Fibrosis Is Associated With Diastolic Dysfunction in HFpEF. JACC Cardiovasc Imaging 2014

6. Tseng WY, Liao TY, Wang JL. Normal systolic and diastolic functions of the left ventricle and left atrium by cine magnetic resonance imaging. J Cardiovasc Magn Reson 2002; 4:443-457

7. Nagatomo Y, Carabello BA, Coker ML, et al. Differential effects of pressure or volume overload on myocardial MMP levels and inhibitory control. Am J Physiol Heart Circ Physiol 2000; 278:H151-161

8. Cheng WP, Wang BW, Chen SC, et al. Mechanical stretch induces the apoptosis regulator PUMA in vascular smooth muscle cells. Cardiovasc Res 2012; 93:181-189

9. Kim H, Lee J, Hyun JW, et al. Expression and immunohistochemical localization of galectin-3 in various mouse tissues. Cell Biol Int 2007; 31:655-662

**Supplemental Figure Legends**

**Supplemental Figure 1**. After 2 weeks of aortic banding, the tissue Galectin-3 also increases significantly. Cropped western blots were compared between controls and aortic banding animals for Galectin-3 protein concentrations (Figure 2D). Full-length blots are included in Supplemental Figure 1.

**Supplemental Figure 2**. Receiver-operating-characteristics (ROC) analysis of Galectin-3 (A), N-terminal of the prohormone brain natriuretic peptide (B) and left ventricular mass/fibrosis (C) for differentiation mild from severe diastolic heart failure. AUC, area under curve is 0.87, 0.64, and 0.82 for Galectin-3, N-terminal pro-brain natriuretic peptide (NT-proBNP), left ventricular (LV) mass/fibrosis respectively.

**Supplemental Figure 3**. The time courses of the left ventricular volume (left) and the rate of volume change (right). The normalized end-systolic volume (nESV) and end-diastolic volume (nEDV) are determined by the minimal and maximal values from the volume time curve (left). From the rate of volume change curve (right), the minimum and maximum values are identified as the peak ejection rate (PER) and peak filling rate (PFR). The time for deceleration (Tdec) is the time interval between PFR and the zero intercept of the deceleration slope.

**Supplemental Table 1.** Proportion of Grade Diastolic Function According to Criteria in the American Society of Echocardiography Guidelines

| Criteria | Mild/severe DHF |
| --- | --- |
| Mitral E/A ratio | 126/20 |
| Deceleration time (msec) | 120/26 |
| Averaged E/e′ (cm/sec) | 112/34 |

DHF, diastolic heart failure; E, early mitral valve flow velocity; A, late mitral flow velocity; e’, early diastolic lengthening velocities in tissue Doppler imaging

Supplemental Figure 1


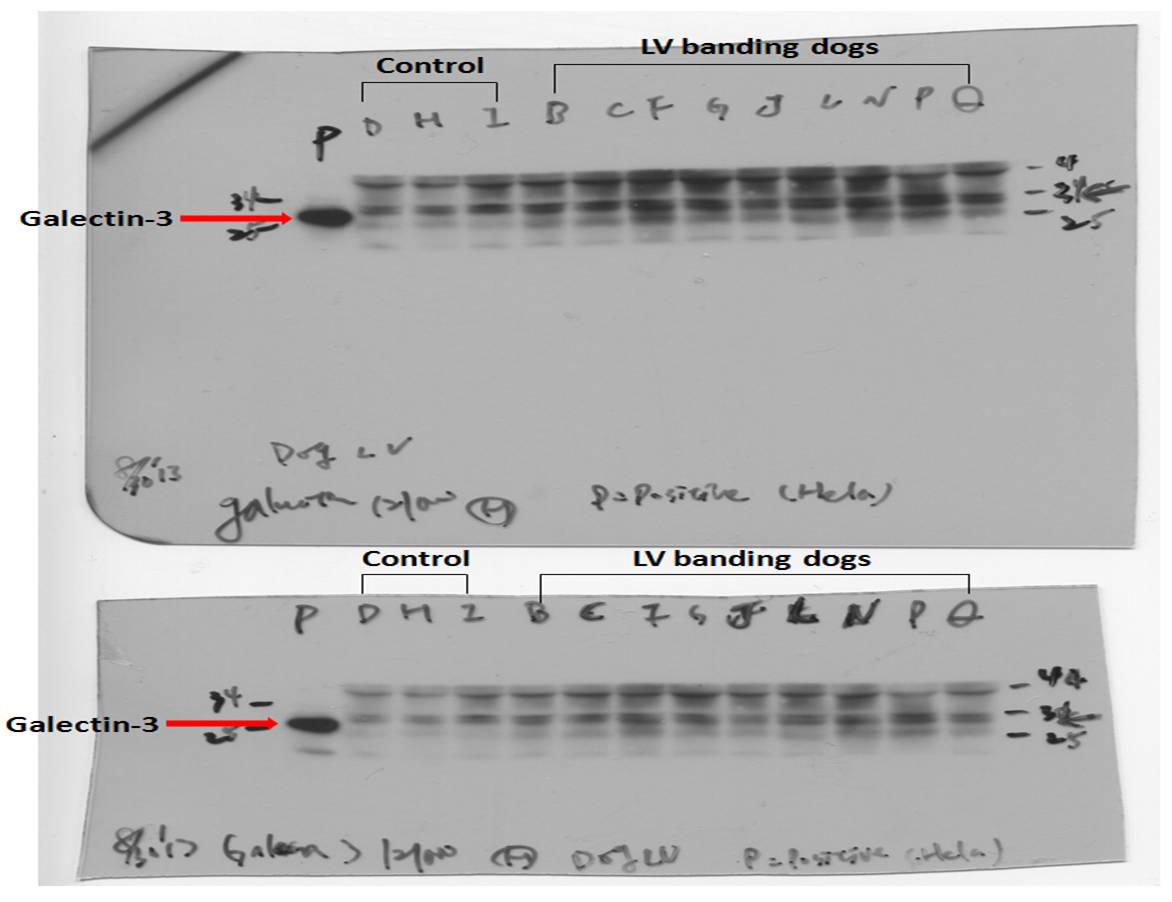


Supplemental Figure 2
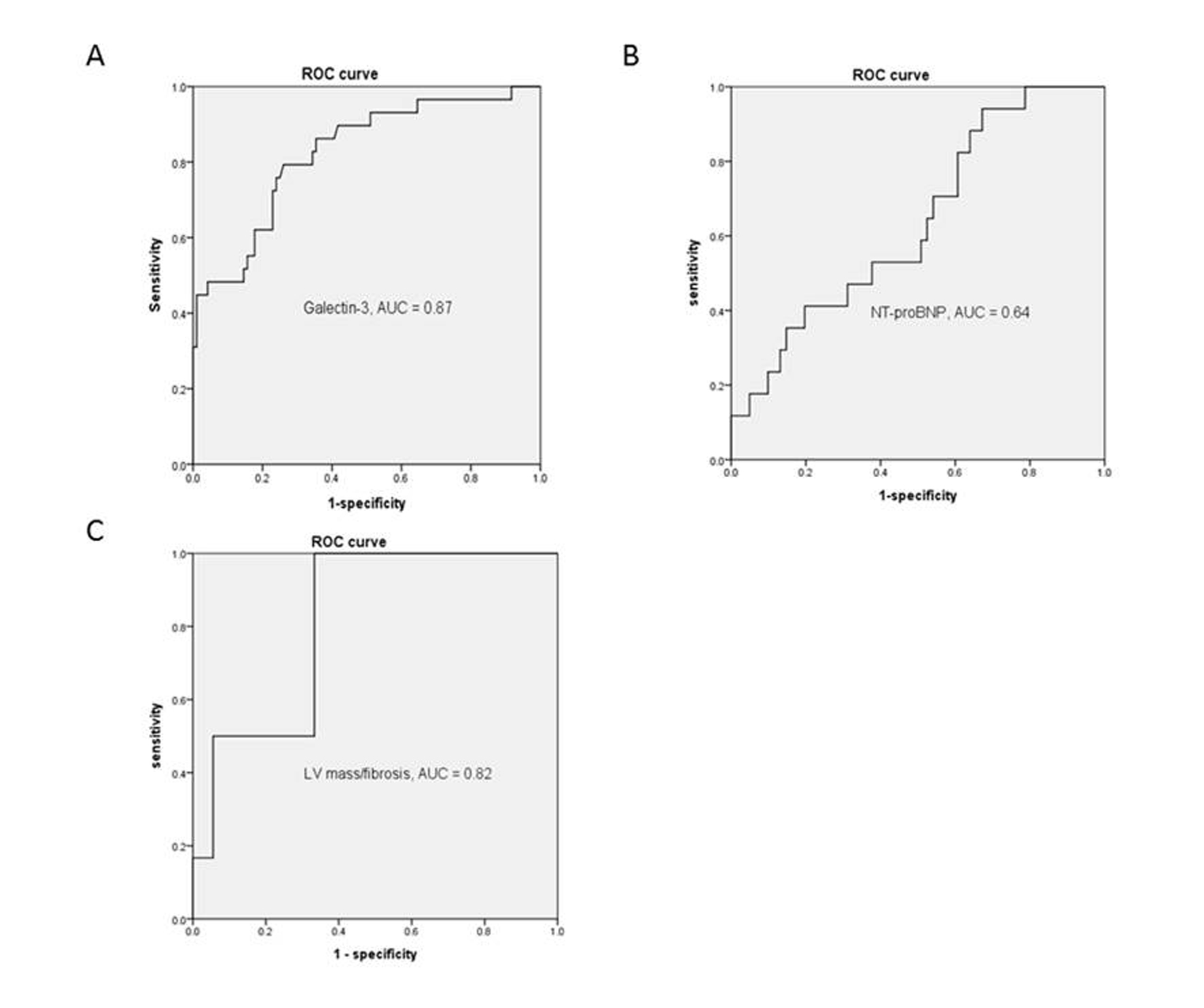
\

Supplemental Figure 3


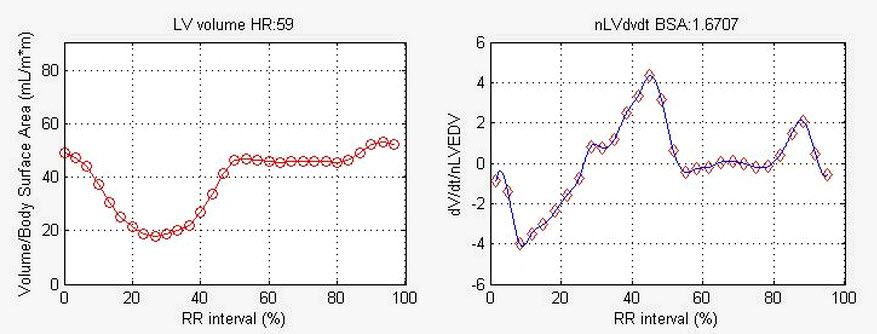

Supplement: Supplementary Information [file srep17007-s1.doc]
